# Sepsis and Nosocomial Infection: Patient Characteristics, Mechanisms, and Modulation

**DOI:** 10.3389/fimmu.2018.02446

**Published:** 2018-10-23

**Authors:** Scott J. Denstaedt, Benjamin H. Singer, Theodore J. Standiford

**Affiliations:** Division of Pulmonary and Critical Care Medicine, Department of Internal Medicine, University of Michigan Medical School, Ann Arbor, MI, United States

**Keywords:** sepsis, compensatory anti-inflammatory response, priming, nosocomial infection, immunosuppression, SIRS, immunostimulation

## Abstract

Sepsis is a leading cause of death worldwide. After initial trials modulating the hyperinflammatory phase of sepsis failed, generations of researchers have focused on evaluating hypo-inflammatory immune phenotypes. The main goal has been to develop prognostic biomarkers and therapies to reduce organ dysfunction, nosocomial infection, and death. The depressed host defense in sepsis has been characterized by broad cellular reprogramming including lymphocyte exhaustion, apoptosis, and depressed cytokine responses. Despite major advances in this field, our understanding of the dynamics of the septic host response and the balance of inflammatory and anti-inflammatory cellular programs remains limited. This review aims to summarize the epidemiology of nosocomial infections and characteristic immune responses associated with sepsis, as well as immunostimulatory therapies currently under clinical investigation.

## Introduction

In 1996, following several failed trials aimed at treating the systemic inflammatory response syndrome (SIRS) (1–3), Roger C. Bone first urged a paradigm shift toward understanding the “compensatory anti-inflammatory response (CARS)” in sepsis (4). Decades later, sepsis remains a leading cause of morbidity and mortality affecting over 30 million people worldwide each year (5), yet not a single immune modulating therapy is actively being used in the clinical setting today. Though mortality rates have declined over time with advances in supportive care, improvements in sepsis therapy are needed to combat persistently high mortality (6, 7). The most recent focus has been on delivery of precision medicine through immunomodulation of the altered host response in sepsis.

Early observations of immune dysfunction in the critically ill come from the trauma and surgical literature (8–10). Decreased cytokine responses to stimulation were later identified and associated with decreased survival in patients with septic shock (11). Though a correlation between sepsis and nosocomial infection has been clinically recognized for decades, it is mostly within the last 10–15 years that this clinical entity has been strongly associated with depressed host immune responses. Large observational studies examining the impact of secondary infection on morbidity and mortality in patients with sepsis are limited and at times data are conflicting. Here, we review the evidence for increased susceptibility to secondary infection in sepsis, mechanisms of depressed host immunity, and promising therapies to modulate the host response.

## Epidemiology of nosocomial infection after sepsis

Nosocomial infections after sepsis are common. However, there are wide variations in the reported incidence and associated morbidity. Sepsis-related immunosuppression in the form of depressed cytokine responses and lymphocyte apoptosis has been hypothesized as the main factor contributing to this complication, though evidence supporting causation is found mainly in experimental models of sepsis. A number of small retrospective observational studies have demonstrated increased risk for nosocomial bacterial and fungal infections in patients with sepsis (12, 13). Likewise, reactivation of dormant viral infections has also been well-recognized and occurs concomitantly with other nosocomial infections (14). For the purposes of this review, we will focus on two large observational studies that have examined this topic in detail.

One large retrospective study estimated that 1 in 3 patients with sepsis will develop a nosocomial infection and half of these infections will occur in the lung (15). A larger prospective study found that 1 in 8 patients will develop nosocomial infection and one-quarter of these will be pulmonary infections (16). In both studies, nosocomial infection developed in the late phase of sepsis at a median of 9 days from admission. In the study by Zhao and colleagues, the most common site of secondary infection was pulmonary (52.5%) and there was no association between primary site of infection (e.g., pulmonary, abdominal, skin/soft tissue, urinary) and the development of secondary infection. In the study of van Vught and colleagues, the most common site of secondary infection was cardiovascular (35.3%). The distribution of primary and secondary infection sites in both studies were distinct, suggesting that secondary infection resulted from a new infectious insult rather than inadequately managed primary infection. Patient risk factors for development of nosocomial infection were similar and included older age, higher illness severity score, longer intensive care unit (ICU) length of stay (LOS), and respiratory insufficiency. ICU-specific exposures such as central venous catheterization and endotracheal intubation also increased risk. The most common causative pathogens were bacterial in both studies. As list of typical sites of nosocomial infection and pathogens are shown Table 1.

**Table 1:** Primary and secondary sites of infection and etiology of secondary nosocomial infection in patients presenting with sepsis.

**Infection site**	**Primary**	**Secondary**
Pulmonary	48%	25.4%
Cardiovascular*	7.3%	35.3%
Abdominal	19%	15.9%
Neurological	2.2%	12.7%
Skin/Soft tissue	2.2%	3.9%
Urinary	4.3%	1.2%
Other^¥^	16.8%	19%
**TYPICAL NOSOCOMIAL PATHOGENS**		
**Gram positive (45.2%)**		
• *Staphylococcus epidermidis* (14.7%)		
• *Enterococcus faecalis* (12.0%)		
• *Enterococcus faecium (*6.3%)		
• *Staphylococcus aureus (*6.0%)		
• Others (6.2%)		
**Gram negative (26.6%)**		
• *Pseudomonas aeruginosa* (9.0%)		
• *Escherichia coli* (3.9%)		
• *Klebsiella pneumonia* (2.7%)		
• *Stenotrophomonas maltophilia* (2.7%)		
• Others (8.3%)		
**Fungi (9.6%)**		
• *Candida albicans* (2.7%)		
• *Candida glabrata* (1.2%)		
• Others (5.7%)		
**Viruses (9.9%)**		
• *Herpes simplex* (3.9%)		
• Cytomegalovirus (2.1%)		
• Others (3.9%)		

Data from Van vught et al. (16) Table 2 and Supplementary Table 3. ^*^Cardiovascular site of infection included bacteremia and catheter-related bloodstream infections ^¥^Other sites of infection included lung abscess, sinusitis, pharyngitis, tracheobronchitis, endocarditis, mediastinitis, myocarditis, postoperative wound infection, bone and joint infection, oral infection, eye infection, reproductive tract infection.

Nosocomial infection was associated with increased hospital LOS in both studies, but the effect on mortality varied between 15 and 21%. The adjusted absolute increase in mortality specifically attributable to nosocomial infection (population attributable mortality fraction) was only 2% (16). These data suggest that a significant portion of the difference in mortality after sepsis is actually due to competing factors such as higher admission illness severity rather than nosocomial infection. Other studies have made similar observations linking illness severity to outcome, rather than nosocomial infection (17). Furthermore, critically ill patients without sepsis had similarly high rates of nosocomial infection suggesting that ICU exposure, rather than sepsis itself, contributes largely to the development of nosocomial infections. However, infections in patients with sepsis were more commonly due to opportunistic pathogens (enterococci, *Pseudomonas aeruginosa* and viruses) implying there still may be a link to sepsis-related immunosuppression.

Both exposures and host susceptibility play a role in development of nosocomial infection. As such, differences among studies in nosocomial infection and mortality rates are likely due to differences in patient selection, ICU type, primary type of sepsis, infectious diagnostics/definitions, infection prevention practices, and geographical location. Regardless of their impact on mortality, nosocomial infections are common and remain a significant factor in morbidity during recovery from sepsis. In addition, they are a burden on the health care system and account for an additional $20,000–40,000 dollars per episode (18). Whether immunostimulatory therapies will reduce rates of secondary infection in patients with sepsis will be determined in ongoing clinical trials.

## Does the biphasic model explain the heterogeneity of response in sepsis?

### The biphasic model

**Figure 1:**
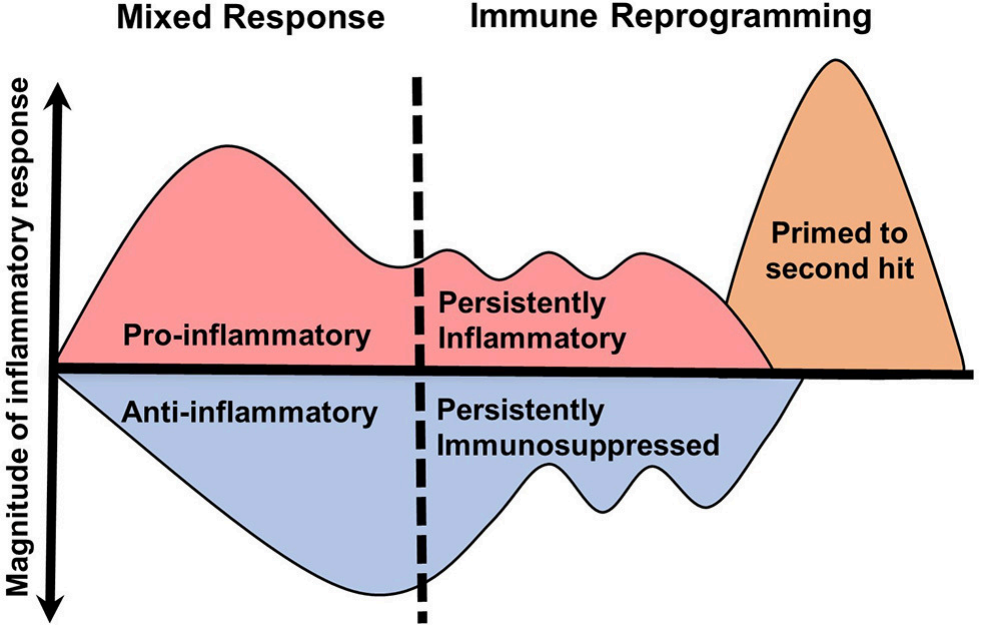
Revised model of inflammation in sepsis. The traditional biphasic model of sepsis (19) plots the immune system on a timeline with an initial hyperinflammatory cytokine storm followed by hypoinflammatory immune paralysis. However, clinical evidence does not support well-demarcated immune phases. In this revised model, the initial immune response to sepsis is a continuous mix of pro- and anti-inflammatory processes that lead to specific immune reprogramming. These programs include persistently pro- or anti-inflammatory and primed responses. The duration and magnitude of each inflammatory program is likely result of many determining factors.

The biphasic model of sepsis has been hypothesized for nearly two decades (19). This model depicts an initial hyperinflammatory response followed by prolonged immune paralysis resulting in morbidity and mortality. However, it is well recognized that the septic immune response does not fit a linear timeline of enhanced inflammation with progression to impaired immunity. Evidence to support this comes from multiple studies, including systematic reviews of gene expression microarray data from blood leukocytes over the course of sepsis (20). In this study, no clear immunosuppressive phase was identified. In fact, at any given timepoint pro- and anti-inflammatory genes were expressed simultaneously in the same patient. Similarly, others have demonstrated circulating anti-inflammatory mediators, such as IL-10, in conjunction with prototypical inflammatory cytokines (TNFα, IL-6, IL-8) at the onset of septic shock (21, 22). Sepsis is therefore a heterogeneous continuum of pro and anti-inflammatory immune programs occurring concurrently. There is also evidence supporting primed immune programs, discussed later in this section, that may occur during the course of sepsis or recovery. A revised model of the inflammation is therefore necessary to illustrate the simultaneous nature of these processes (Figure 1).

To complicate matters further, cytokines often do not behave in a dichotomized manner (23). A single cytokine may contribute to survival or death depending on the context in which it is examined (23). For example, IL-10 neutralization 24 h after cecal ligation and puncture (CLP) is protective against secondary *Pseudomonas aeruginosa* pneumonia (24). However, IL-10 neutralization performed prior to endotoxemia or CLP (25, 26) is lethal. A cytokine may also act pleiotropically depending on the environmental context. For instance, IL-10 has suppressive and priming effects depending on the adherent state of a monocyte (27). Non-adherent monocytes are actually primed by this “anti-inflammatory” cytokine to produce more TNFα, IL-6, and IL-1ra upon endotoxin challenge *ex vivo*. The net effect of an inflammatory mediator is therefore highly contextual.

Moreover, the inflammatory response is tissue-specific (28). Differential expression of inflammatory mediators has been noted in mouse tissues in response to systemic endotoxin. IL-1α production is maximally increased in lung, spleen and liver, while IL-6 is increased in the heart, muscle, brain and kidney (29). In a rat model of CLP examining the molecular response to sepsis, though a subset of gene expression was shared, each organ had a distinct molecular fingerprint (30). Tissue-level responses to secondary stimulation may also be discrepant in the septic host. For example, hemorrhage prior to CLP in mice caused primed responses in alveolar macrophages and Kupffer cells, whereas splenic macrophages and peripheral blood mononuclear cells demonstrated decreased cytokine production consistent with endotoxin tolerance (31). The magnitude of response to an inflammatory stimulus is also variable, as *in*-*vivo* imaging of lipopolysaccharide (LPS)-induced NF-κB activation has shown heterogenous intracellular activation more prominently in the skin, lungs, spleen, and small intestine as compared to other organs (32). These observations illustrate two important conceptual points: (1) the (tissue) compartmental response to sepsis and other inflammatory stimuli is highly variable both in quality and magnitude and (2) primed and suppressed responses may be present simultaneously within the same organism following a single exposure. These experimental studies raise important questions that require further investigation in humans.

### Priming

Primed immune responses in sepsis may contribute to the heterogenous inflammatory response and are not accounted for in the biphasic model. Priming requires an initial exposure to the host that results in an enhanced inflammatory response to secondary stimulation. For example, Kupffer cells are primed to produce more TNFα in response to endotoxin when femur fracture precedes CLP (33). Similarly, hemorrhage prior to CLP enhances production of plasma IL-6 and TNFα (31). Priming during sepsis may be a protective mechanism, as seen in a model of enteral *Enterococcus faecalis* infection (34). In this study, mice were pre-exposed to mild or severe sterile systemic inflammation using varying degrees of pancreatitis or thermal injury. Mice experiencing mild inflammation were protected from *E. faecalis* related mortality, this was associated with primed IL-12 production and enhanced phagocytic function in peritoneal macrophage.

Priming may also represent a mechanism of late organ injury in survivors of sepsis. It is well recognized that survivors of sepsis are at increased risk for long-term cognitive impairment (35), new cardiac events (myocardial infarction, cerebrovascular accident, sudden cardiac death) (36), and new renal failure (37). The cause of these complications remains unclear, though persistent inflammation and primed immune responses have been hypothesized to contribute. Murine CLP models have demonstrated enhanced TNFα production in splenic inflammatory monocytes and brain microglia for at least 2 weeks after sepsis (38, 39) suggesting a possible link between primed cells and long-term organ dysfunction. Meanwhile, persistent inflammation may influence patient outcome, as observations of persistent elevation of IL-6 in patients with pneumonia have been associated with increased risk for long-term mortality due to cardiovascular disease or renal failure (40). Similarly, models of sepsis survival have demonstrated progressive atherosclerosis in the setting of low-grade circulating inflammation (41) and neurocognitive dysfunction associated with persistent neuroinflammation (38, 42) weeks to months after polymicrobial sepsis. In a mouse model of pneumococcal pneumonia, recruitment of inflammatory monocytes to the brain was associated with microglial activation and long-term cognitive impairment (43). When monocyte recruitment was abolished, neuroinflammation was reduced and cognitive impairment was improved. While these initial findings are exciting, the relationship between persistent inflammation, immune priming and long-term organ injury needs to be understood in more detail.

**Figure 2:**
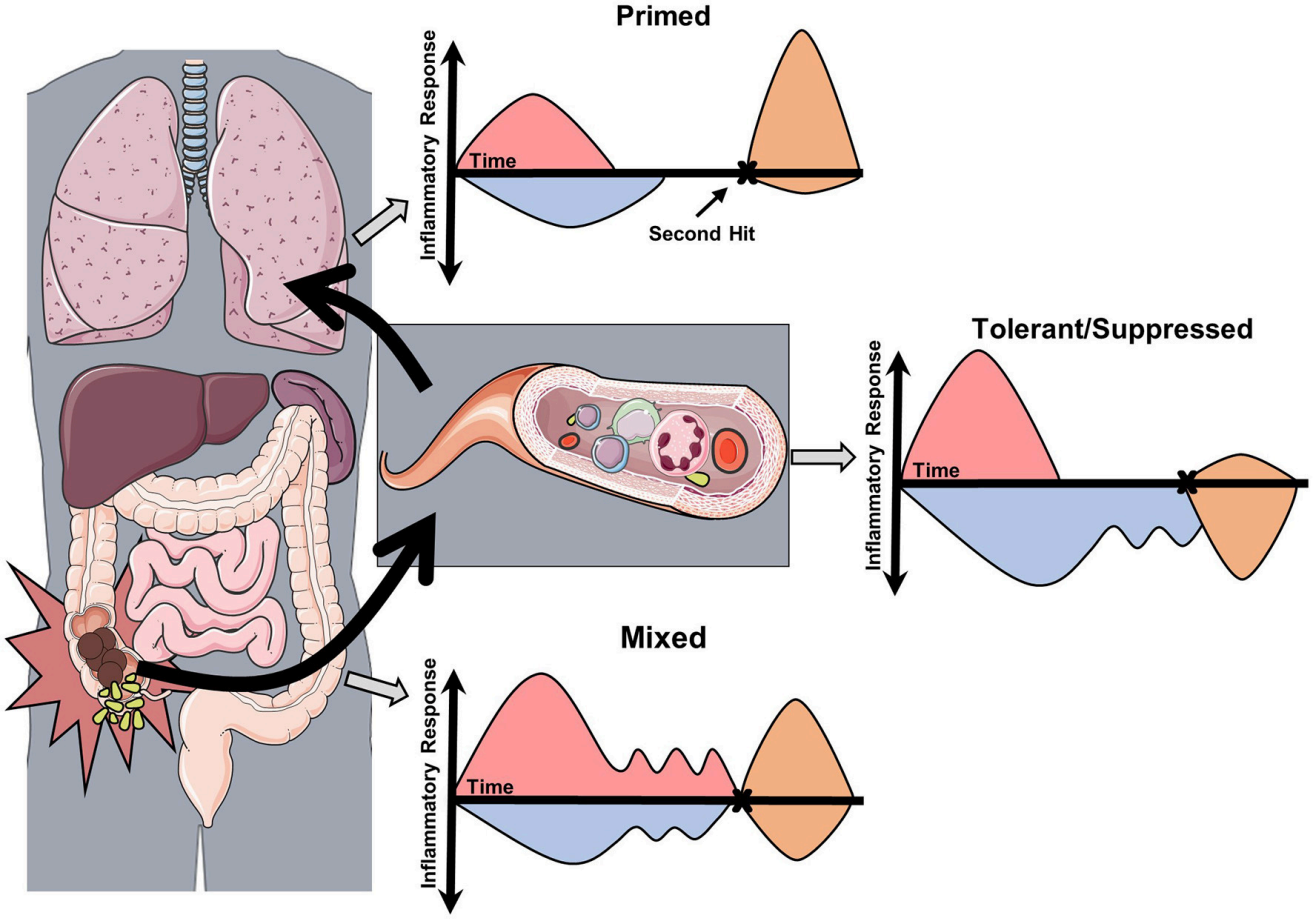
Conceptual model of the compartmentalization and heterogeneity of sepsis. This conceptual model is derived from studies in experimental sepsis that have demonstrated tissue-specific inflammatory responses. In this model, acute sepsis in one compartment (abdomen) leads to specific and dynamic changes in proximal (blood) and distal (lungs) compartments. Assessment of the immune response by *ex vivo* stimulation assays (second hit) may then reveal the predominant cellular program. In this case, each compartment responds differently to secondary stimulation based on the severity and composition of the preceding inflammatory insult.

**Figure 3:**
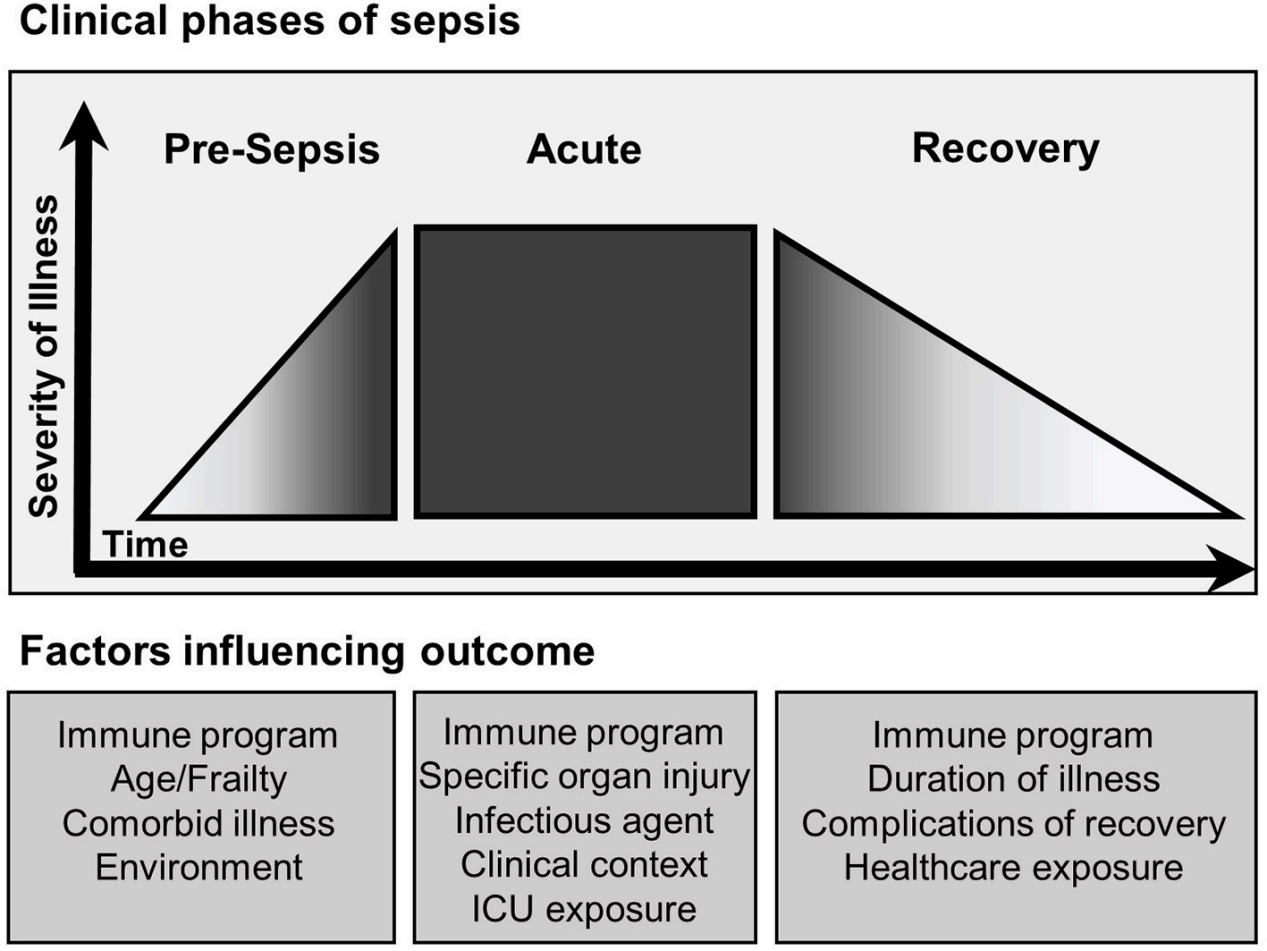
Clinical phases of sepsis and factors influencing outcome. The clinical course of sepsis is characterized by accelerated progression in severity of illness leading to the development of clinical sepsis. The outcome of each clinical phase of sepsis is influenced by multiple factors. The pre-sepsis phase is influenced primarily by the baseline functional state of the patient. Pre-sepsis functionality directly affects the course of acute sepsis including onset, magnitude and duration. Furthermore, properties inherent to the type of sepsis and exposures occurring during management of acute sepsis continue to affect outcome. Recovery follows and is largely dependent on the severity of prior phases, though continued exposure to the healthcare system places patients at risk for nosocomial complications. Throughout each phase the specific immune program is heterogenous and influences outcome.

There is strong experimental evidence supporting the conclusion that the inflammatory response of sepsis is heterogeneous at a molecular, cellular, tissue compartment, and individual level. Though further studies are needed in human sepsis, the possibility of “within patient” compartment specific immune heterogeneity warrants consideration. A new conceptual model of the patient experiencing sepsis is required (Figure 2), as one may have reduced immune response on peripheral blood assays, yet in other compartments the net immune response may be mixed or primed. With the introduction of immune stimulating therapies, one must consider that disproportionately primed organs may be harmed by this therapy. Decisions on how to modulate the immune response may be informed by *ex vivo* stimulation assays, but consideration should be taken to survey for multiple cellular programs in multiple tissue compartments. In addition, these programs are not limited to the acute phase of sepsis, as immune reprogramming influences the entire clinical course including recovery (Figure 3). Pre-sepsis immune status is likely to be an important determinant of which predominant cellular program manifests during acute illness. Post-sepsis immune status may also be impacted by the preceding phases of illness. Likewise, chronic comorbidities may influence the magnitude and evolution of both responses. Despite the evidence supporting sepsis-related immunosuppression in this review, there are large pieces of the immune response to sepsis that remain a mystery and our understanding of sepsis heterogeneity is only in its infancy.

## The immunosuppressive cellular program of sepsis

**Figure 4:**
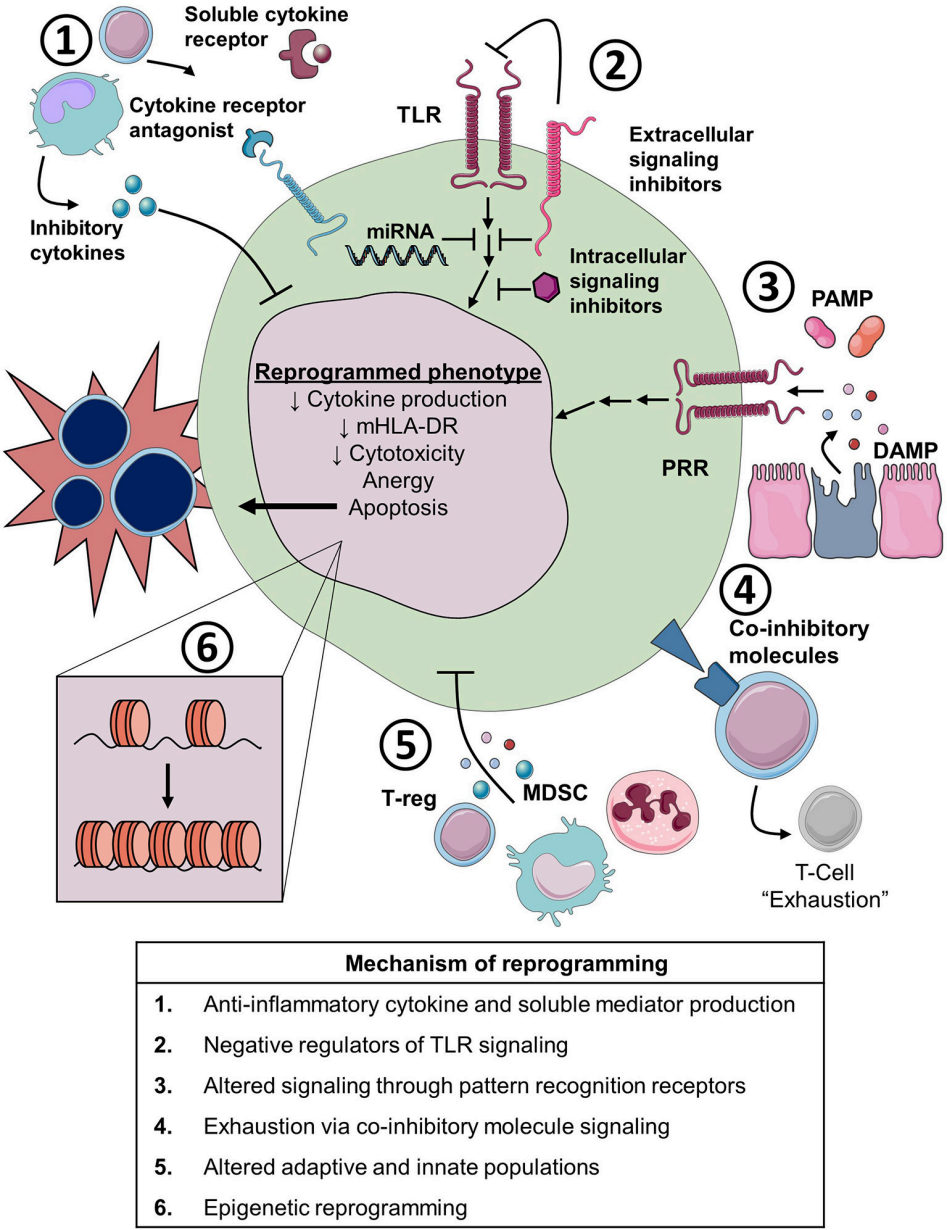
Cellular and molecular mechanisms of immune reprogramming in sepsis. TLR, toll-like receptor; miRNA, microRNA; PAMP, pathogen associated molecular pattern; DAMP, damage associated molecular pattern; PRR, pathogen recognition receptor; MDSC, myeloid derived suppressor cell; T-reg, regulator T-cell; mHLA-DR, monocyte Human Leukocyte Antigen-DR.

Critically ill patients with sepsis, trauma, and burns experience similar immunosuppressive phenotypes. Broadly, these include enhanced cellular apoptosis, suppressed cytokine production, decreased major histocompatibility complex (MHC) class II surface markers, reduced antigen presentation, anergy, and diminished cytotoxic effector cell function. Collectively, these septic leukocyte responses are known as the immunoparalysis or immune exhaustion. While these phenotypes may be associated with increased risk of nosocomial infections and death, they are not inclusive of broader immune changes in sepsis such as potentially primed cells. Moreover, though an exhausted cell may down-regulate cytokine responses, other cellular functions may be simultaneously upregulated (44, 45). As such, the term “cellular reprogramming” is more appropriate to describe general immunophenotypic changes occurring during sepsis. Several examples of immunosuppressive cellular program are described below and are summarized in Figure 4.

### Cellular apoptosis

Apoptosis of T and B lymphocytes has been demonstrated in models of sepsis (46), post-mortem analysis of septic patients (47–49), and in the circulation of patients with septic shock (50). Resultant lymphopenia is associated with increased mortality and risk of nosocomial infection (50, 51) and occurs commonly in patients with persistent critical illness (52). Enhanced apoptosis also occurs in myeloid and epithelial cells including blood monocytes (53, 54), dendritic cells (55), intestinal (48) and pulmonary epithelial cells (56), but not neutrophils (57). Apoptosis of monocytes may be a protective mechanism, as acute apoptosis of these cells has been associated with improved mortality (54). Broad reversal of lymphocyte apoptosis through caspase inhibition or over-expression of the antiapoptotic B-cell lymphoma 2 (Bcl-2) protein in experimental sepsis has subsequently been shown to improve mortality (58, 59). As such, improving sepsis-induced lymphocyte apoptosis and lymphopenia has been a primary target for immunomodulation.

### Suppressed cytokine release

Immunodepression, immune tolerance, and immunoparalysis are all terms used to describe the decreased production of various cytokines, including TNFα, IL-1β, IL-6, and IFNγ, after *ex vivo* stimulation of leukocytes with endotoxin or other pattern recognition receptor (PRR) agonists. This phenotype in sepsis is similar to the *in vitro* phenomenon of endotoxin tolerance, whereby stimulating with high concentration endotoxin results in decreased responses upon secondary stimulation. In sepsis, decreased cytokine production in response to endotoxin stimulation has been demonstrated in whole blood (60), peripheral blood mononuclear cells (61), adherent monocytes (11), neutrophils (62), and NK cells (63). This phenotype occurs early in the course of sepsis and resolves in survivors. However, failure to resolve immune tolerance is associated with increased mortality (11), and expression of endotoxin-tolerant gene signature has been associated with sepsis induced organ failure (64). Though immune tolerance is associated with increased risk of nosocomial infection in trauma patients (65), the association in sepsis is less clear (66, 67).

### Alterations in HLA-DR expression and other surface markers

Changes in cellular surface markers occurs during sepsis, with reduced expression of MHC class II molecules on monocytes being the most well studied. Low monocyte human leukocyte antigen (mHLA)-DR correlates with endotoxin tolerance and suppression of antigen-specific T cell responses (68). Early studies found increased rates of sepsis in trauma patients with low mHLA-DR expression (69). Subsequently, low mHLA-DR on admission for sepsis or septic shock has been associated with increased mortality (70, 71) and nosocomial infections (72). Failure to restore mHLA-DR expression over the course of illness is also associated with worse outcome in patients with severe sepsis (66). While highly predictive of mortality and nosocomial infection, mHLA-DR expression dynamics are contextual and dependent on the infectious agent (73) with Gram-positive infections showing lower monocytic mHLA-DR expression than Gram-negative bloodstream infections. The reliability of mHLA-DR to predict outcome and its dynamics of expression are currently under investigation as part of two large observational cohort studies (74, 75). Neutrophil surface markers are also altered, including CD88 expression which is associated with reduced phagocytic function and increased risk for nosocomial infection (76). Importantly, the presence of multiple surface marker abnormalities (low mHLA-DR, low neutrophil CD88 and increased T regulatory cell markers) was most associated with nosocomial infection than any single marker alone (77).

### Gene expression endotypes

Functional assays and cell surface marker assessments remain the standard method to assess immunosuppression in sepsis. However, whole-genome expression endotypes correlating with survival of sepsis have been discovered using advanced statistical techniques (78, 79). Davenport et al. found increased 14-day mortality and illness severity associated with a gene expression endotype that was characterized by functional changes in T cell activation, apoptosis, endotoxin tolerance, and down-regulation of HLA class II molecules. Variation in genomic DNA sequence was also associated with specific gene expression patterns, supporting a genetic mechanism for individual heterogeneity. In a follow-up study of patients with community-acquired pneumonia (CAP) and fecal peritonitis (FP), the genomic response to sepsis was largely similar between types of infection with only a modest number of genes differentially expressed between CAP and FP (80). Serial sampling over the course of sepsis demonstrated that patients may switch between the defined endotypes during the course of disease. These studies confirm what is known about clinical sepsis, that phenotype is both heterogenous and dynamic. Classification of patient immune responses quickly and dynamically remains a priority for precision medicine in sepsis. In addition, while mixed leukocytes in whole blood have been reliably used for gene expression analysis, cell-type specific gene expression in sepsis may reveal additional endotypes and help us understand therapies that may reliably be used to modify them.

### Lung-specific changes in immunity

The lung is uniquely and continuously exposed to the external environment and acts as a first line of defense against environmental pathogens, especially in critically ill patients with respiratory failure requiring mechanical ventilation. Consequently, the lung is a primary site for nosocomial infection in patients with sepsis. The alveolar macrophage (AM) represents the predominant immune effector cell in the alveolus. Similar to the dysfunction of blood monocytes, decreased TNFα production in AM of mice and humans with sepsis has been recognized for quite some time (81). In polymicrobial sepsis models, AM display both depressed cytokine responses to endotoxin challenge and decreased phagocytic capacity (82). Neutrophil recruitment to the alveolus is decreased (83) and recruited neutrophil ROS production is depressed (84). In an *Escherichia coli* pneumonia model, lung parenchymal dendritic cells demonstrated decreased antigen presenting capacity and reduced immunostimulatory responses during recovery from sepsis (85). Moreover, these depressed responses were specific to the pulmonary compartment and were mediated by local inflammatory factors released upon organ injury. Disruption of the epithelial barrier leads to alveolus permeability, leak, and decreased mucocilliary clearance all of which may predispose to development of nosocomial infection (86). General loss of pulmonary epithelial barrier function is noted with pulmonary epithelial cell apoptosis in polymicrobial sepsis models (87) and in patients with acute lung injury due to sepsis (56).

### Anergy and cytotoxicity

Anergy due to a failure of T cell proliferation or elaboration of cytokines in response to specific antigens has also been described. An increased risk of post-operative sepsis and death was initially described in patients with anergy to delayed-type hypersensitivity skin testing (88). Similarly, patients with lethal post-operative peritonitis had reduced T cell proliferation and secretion of both IL-2 and TNFα in response to CD3/CD28 cross-linking (89). In sepsis models, development of anergy is mediated via a population of TNF-related apoptosis-inducing ligand (TRAIL) expressing CD8^+^ T cells (90). In humans, CD4^+^, CD25^+^, CD127^lo^ regulatory T cells (Treg) have been associated with reduced mitogen responses and development of anergy (91). Depressed cytotoxic responses have been reported in various cell types. Impaired NK cell function with reduced IFNγ secretion and cytotoxicity has been reported in patients with sepsis (92), while others have found normal NK cytotoxic function in severe sepsis (93).

## Mechanisms of altered immunity

There are many potential mechanisms for altered immunity, both suppressed and primed, in patients with sepsis. Here we highlight several important mechanisms with a primary focus on mechanisms of immunosuppression (Figure 4).

### Anti-inflammatory cytokines and soluble receptors

Three decades of research examining the cytokine response in sepsis is too broad a topic to review here and extensive reviews have already been published on the subject (21). Several cytokines and anti-inflammatory mediators are associated with worse outcomes in septic patients. IL-10 suppresses the pro-inflammatory immune response through deactivating innate immune cells (94, 95). IL-10 is elevated early in the course of sepsis (96) and persistent elevations increase risk of death (97). As discussed previously, this cytokine has pleiotropic roles in experimental models of sepsis, though it may have a particular importance in sepsis-induced impairment of lung immunity (24). IL-10 is actively secreted by multiple cell types that are expanded in septic patients including Treg and myeloid derived suppressor cells (MDSC), which are discussed in more detail below. Though IL-10 has direct immunosuppressive effects, its association with development of nosocomial infection is less straight forward (96). Enhanced IL-10 signaling has been associated with the development of nosocomial infection in at least one study (98).

Soluble receptors for cytokines are additional anti-inflammatory mediators that have been long recognized in sepsis. These molecules are shed cell-surface receptors that neutralize the activity of pro-inflammatory cytokines and are largely viewed as a protective mechanism. TNF soluble receptors I and II (sTNFR-I, sTNFR-II) levels are increased in septic patients and are associated with mortality (97, 99, 100). Though there is minimal data linking sTNFR to nosocomial infections in sepsis, they are chronically elevated in the elderly and therefore elevated levels may represent a predisposition to infection (101).

IL-1 receptor antagonist (IL-1ra) is a naturally occurring antagonist to IL-1. IL-1ra levels are significantly elevated in patients with septic shock (97, 102) and are associated with increased mortality (103). Recent retrospective analysis of the IL-1 pathway in a previously completed trial of anti-IL1 therapy in sepsis showed a mortality benefit of anti-IL1 antibody administration in patients with the highest levels of circulating IL-1ra (104). These data suggest that the levels of soluble receptors and receptor antagonists may be markers of mortality by indicating the magnitude of the pro-inflammatory response. In addition, recent data have suggested a link between an initial dysregulated hyperinflammation and subsequent development of nosocomial infection (98). Gene expression analysis of leukocytes from patients developing nosocomial infection has demonstrated overactivation of IL-1 signaling (16) supporting a potential relationship between elevation of IL-1ra and nosocomial infection in sepsis.

### Pathogen recognition receptor signaling inhibitors

The pro-inflammatory host response to microbial mediators occurs through PRRs including the Toll-like receptor (TLR) family. Negative regulators of TLR signaling are induced during sepsis. These regulators selectively inhibit the downstream inflammatory response via interactions with one or multiple TLR pathways. Single immunoglobulin IL-1R-related protein (SIGIRR) interferes with binding of IL-1 and LPS extracellularly and interferes with complexing of IRAK-1 and TRAF-6 intracellularly, resulting in profound effects on NF-κB and MAPK-dependent signaling (105). MyD88 short (MyD88s) splice variant is upregulated in response to LPS and is defective in its ability to phosphorylate IRAK resulting in reduced NF-κB activation (106). Both SIGIRR and MyD88s expression were found to be elevated in septic monocytes and associated with depressed cytokine responses (107). Interleukin-1 receptor associated kinase-M (IRAK-M, also known as IRAK-3) negatively regulates TLR signaling through inhibiting the dissociation of IRAK-1 from the Toll-IL-1 signaling domain. In experimental sepsis, IRAK-M is upregulated in alveolar macrophages and mediates supressed cytokine responses and impaired clearance of *P. aeruginosa* (108). IRAK-M is also elevated in monocytes from septic patients (109). MicroRNAs (miRNA) are small non-coding RNA and have been found to exert negative regulatory effects on TLR signaling. Multiple miRNAs are dysregulated in sepsis. In particular, elevated circulating levels of miRNA 155 have been associated with poor outcome and expansion of regulatory T cells in patients with sepsis (110) indicating a possible link to sepsis immunosuppression and nosocomial infection.

### Pathogen associated molecular patterns

Pathogen associated molecular patterns (PAMPs) are exogenous microbial factors derived from infectious organisms that activate PRRs. In sepsis, PAMPs, such as cell wall and intracellular microbial components, are the primary factors initiating the inflammatory response. PAMPs are therefore critical to the reprogramming of immune cells in sepsis, this reprogramming is likely dependent on the specific antigen (PAMP) and receptor (PRR) combinations that are engaged on a particular cell. For example, *in vitro* stimulation of human monocytes with various PAMPs has demonstrated that the fungal cell wall component β-glucan induces primed (trained) responses through nucleotide-binding oligomerization domain-like receptors (NLRs) (111). In contrast, engagement of PAMP-TLR combinations, such as LPS with its receptor Toll-like receptor 4 (TLR4), induced predominantly tolerant programs with depressed cytokine production. Interestingly, the TLR ligands administered at low dose caused primed responses while inducing tolerance at higher doses, suggesting the presence of an inflammatory rheostat guiding secondary responses. In the context of sepsis, pathogen specific ligands such as LPS (Gram-negative organism) or lipotechoic acid (Gram-positive organism) and endogenous PRR ligands (discussed in the next section) form a complex network of PRR signaling that is likely to contribute to the inflammatory cellular program. In addition to exposure to infectious pathogens, sepsis and critical illness are associated with collapse of the host microbial community, a term known as dysbiosis. This occurs through a combination of ecological factors that are drastically altered in the critically ill (112). While there is no direct evidence linking the dysbiosis that occurs in septic patients to subsequent nosocomial infection, population level studies have demonstrated an increased risk of severe sepsis within 90 days following hospitalizations known to result in dysbiosis (113). A second study demonstrated an increased risk of severe sepsis and septic shock in the 90 days following a hospital-related antibiotic exposure (114). These studies suggest PAMP-PRR interactions via primary infection or continued dysbiosis may promote changes in the immune program that predispose critically ill patients to secondary infection and sepsis, although further investigation is required to establish causal relationships.

### Damage-associated molecular patterns

Damage-associated molecular patterns (DAMPs) are endogenous pattern recognition receptor agonists that initiate inflammatory responses but have distinct biological roles in non-inflammatory states. These proteins are released upon host injury, either passively from necrotic cells or actively secreted into the extracellular space (115). DAMPs are released during injured states, including sepsis, trauma, and burns. As such, DAMPs are appealing candidates as mediators of altered immune programs observed in these patients. Several DAMPs have been shown to correlate with sepsis morbidity and mortality including S100A8/A9, high-mobility group box-1 (HMGB1), mitochondrial DNA, nuclear DNA, histones and heat shock proteins (HSP) (115). HMGB1 and S100A8/A9 are acutely elevated in patients presenting with sepsis and are associated with worse outcome (116, 117). Their functions are pleiotropic, including induction of immunosuppressive MDSC expansion and priming of immune cells in sepsis models (38, 39, 118). HMGB1 also stimulates the expansion of Treg in chronic inflammatory diseases (119). HSPs are intracellular molecular chaperone proteins that can have anti-inflammatory properties. Several HSPs (HSP27, 60, 70, 90) are increased in patients with sepsis and are associated with enhanced neutrophil oxidative activity and reduced apoptosis (120). HSP70 levels increase with the degree of hyperinflammatory response and are associated with increased risk of mortality in patients with sepsis (121, 122). HSP70 also promotes adaptive immune dysfunction through enhanced Treg suppressor activities and secretion of anti-inflammatory cytokines (123). In many of the studies referenced elevated levels of certain DAMPs persist in the circulation of survivors for many days, indicating a potential for ongoing modulation of the immune system after acute sepsis has resolved.

### Expansion of regulatory T cells

Cellular subsets that have roles in homeostasis are expanded during sepsis and may contribute to nosocomial infection susceptibility. Treg are a T cell population that are able to negatively regulate the adaptive and innate immune response (124). There are several subsets of Treg which can be identified by cell surface markers. The CD4^+^, CD25^+^, CD127^lo^ subset is one of several studied in sepsis. These cells are expanded in patients with sepsis (125) and contribute to lymphocyte anergy in septic shock patients (91). Higher Treg numbers are also associated with the development of nosocomial infections in critically ill patients with and without sepsis (77). In sepsis models, Treg expansion results in systemic immunosuppression potentiating tumor growth (126). Data is conflicting though as models of sepsis have demonstrated that antibody mediated depletion of Treg does not improve mortality (127) and adoptive transfer of CD4^+^ CD25^+^ Treg early in the course of sepsis actually improves bacterial clearance and mortality (128). Similarly, in one human study, increased Treg in patients with severe sepsis was associated with improved survival, though this finding may have been driven by higher total T cell counts in these patients (129). Further investigation such as depletion or inhibiting expansion of Treg in humans is required to establish a detrimental role for Treg in patients with sepsis.

### Myeloid-derived suppressor cells

Myeloid-derived suppressor cells (MDSC) are a heterogenous group of immature innate immune cells that exert predominantly suppressive effects. Their expansion is a component of the emergency myelopoietic response to injury, infection and malignancy (130). This population consists of a mix of immature granulocytes, monocytes, and dendritic cells with the ability to suppress T cell function through enhanced arginase, nitric oxide synthase, and reactive oxygen species activity (131). MDSCs can produce a number of pro- and anti-inflammatory cytokines upon secondary stimulation, including IL-10 (132). As such, they represent important effector cells in sepsis recovery. Their release is driven mainly by STAT-3 signaling through inflammatory mediators including IL-6 and colony stimulating factors. Activated STAT-3 also induces release of S100A8/9 which both prevents maturation of MDSCs and promotes additional expansion in a feed forward loop (118, 133). In mouse models of sepsis, MDSCs exert pleiotropic effects, with protective or detrimental properties depending on phase of sepsis in which they are examined (134, 135). MDSCs are expanded acutely in patients with sepsis (136, 137) and are associated with the development of nosocomial infection (138). Their persistence in severe sepsis and septic shock is also associated with increased risk for persistent critical illness and mortality (139).

### Co-inhibitory molecules

Co-inhibitory molecules of the B7-CD28 family function to maintain homeostasis in the host by negatively regulating the immune response (140). In sepsis, these molecules have been postulated to be responsible for immune exhaustion. Post-mortem studies of patients dying of sepsis has demonstrated elevations in co-inhibitory receptors PD-1 and CTLA-4 in splenic T cells, while the ligand PD-L1 was elevated in antigen presenting cells and tissue macrophages (47). T cells and dendritic cells isolated from the lung also expressed increased PD-1 and PD-L1, respectively. Circulating T cells in patients with severe sepsis demonstrated non-statistically significant elevations in PD-1, with a decrease in CTLA-4 (61). Others demonstrated marked elevation in T cell PD-1 and monocyte PD-L1 in septic shock. *In vitro* treatment with anti-PD1 antibody resulted in restoration of monocyte proinflammatory responses and decreased apoptosis of T cells (141). Furthermore, higher monocyte PD-L1 has been associated with mortality, while higher PD-1 and PD-L2 are associated with increased risk of nosocomial infection (142). Others have shown that monocyte PD-L1, and not T cell PD1, expression on day 3–4 of septic shock is an independent predictor of death (143). In mice, neutralization of PD-1 or PD-L1 24 h after sepsis reduced apoptosis of lymphocytes and improved survival (144, 145). In leukocytes isolated from septic patients, *in vitro* blockade of PD1 and PDL1 reverses T cell exhaustion and restores neutrophil and monocyte phagocytic function (146, 147).

### Epigenetic reprogramming

Epigenetic mechanisms of immune dysfunction have also been proposed. Histone modification at inflammatory loci alters the accessibility of DNA to transcription factors and therefore gene transcription. Alterations in chromatin structure are determined by gene activating and repressing histone marks, which are in turn regulated by chromatin modifying enzymes (CME) (148). Specific changes in chromatin are associated with exposure to inflammatory stimuli (149). Not surprisingly, chromatin remodeling occurs in the monocytes of septic patients (150). In models of sepsis survival, dendritic cell cytokine responses were suppressed for up to 6 weeks and were associated with alterations in histone H3 lysine-4 (H3K4) and histone H3 lysine-27 (H3K27) methylation at IL-12 promoter regions. These changes were also mediated by interactions with histone methyltransferases (151). We have demonstrated an IRAK-M mediated reduction in histone H4 acetylation and H3K4 methylation (H3K4me) in immune tolerant AM 24 h after induction of sepsis (152). These studies provide compelling evidence for epigenetic changes in sepsis leading to immunosuppressive phenotypes in both the acute and recovery phases of illness. As such dynamic changes in histones and their CMEs have high potential for use as prognostic markers and therapeutic targets. Epigenetic reprogramming of inflammatory cells may also result in primed phenotypes. This concept is also known as “trained immunity” whereby exposure to specific PAMPs (β-glucan, BCG) leads to specific modifications in chromatin structure and enhanced inflammatory responses to secondary stimulation (153, 154). These modifications include genome wide changes in H3K4me3, H3K4me1, and H3K27 acetylation. Our understanding of trained immunity and its potential implications in the pathophysiology of sepsis-related immune priming remains limited.

## Immunostimulation

Though the immune response to sepsis is dynamic and contextual, there is a large body of evidence supporting an association between immunosuppressive cellular programs and poor outcomes in patients with sepsis. We know that supportive care is inadequate in addressing the complex immunology of sepsis. As such, immunostimulatory therapies have been evaluated to improve sepsis outcomes. Precision approaches in sepsis through immunomodulation require the development of immune monitoring strategies and suitable immunomodulating agents that can be deployed quickly and dynamically at the bedside. The first step is accurately predicting which patients are at risk for secondary infection and mortality related to immune exhaustion. Large observational studies aiming to predict poor outcomes through cellular phenotyping and cell surface marker expression are already underway (74, 75). Given the heterogenous and rapidly evolving immune programs occurring during sepsis, safety and tolerability of immune therapies with a focus on monitoring for hyperinflammatory consequences is essential. Moreover, assessment of long-term outcomes will be important. In addition, recent *in vitro* studies of PD-1/PD-L1 blockade in mononuclear cells from septic patients has demonstrated significant variability in response to immunostimulation (146, 147). Non-response of mononuculear cells to *in vitro* immunostimulation was associated with increased mortality (155). As such, response rates and mechanisms of response will also need to be examined in further detail, similar to the current practices of immune modulation in oncology (156). There are a number of ongoing trials of immunostimulation in sepsis (Table 2).

**Table 2:** Current clinical evidence for immunostimulation in patients with sepsis.

**Therapy**	**Goal of therapy**	**Human Evidence**	**References**
G-CSF/ GM-CSF	Accelerate innate immune cell production Restore mHLA-DR expression and cytokine production	Enhanced resolution of infection^1^ Decreased length of ICU stay^1^ Minimal adverse events^1^ May be delivered directly to lung^2^ Pending results from phase III clinical trial^3^	Bo et al. (157) Scott et al. (158) NCT02361528
IFNγ	Increase phagocytic capacity Restore mHLA-DR expression and cytokine production	Enhanced resolution of bacterial and fungal infection (case series)^1, 2^ Pending results from phase IIIb trial^3^	Nalos et al. (159) Delsing et al. (160) NCT01649921
IL-7	Accelerate lymphocyte production Decrease lymphocyte apoptosis	Well tolerated in phase IIb trial^1^ Increased CD4^+^ and CD8^+^ lymphocytes^1^ Increased T cell activation and trafficking^1^	Francois et al. (161)
Anti-PD-1/ PD-L1	Reverse innate and adaptive immune exhaustion Restore mHLA-DR expression and cytokine production	Well tolerated in patients with sepsis and septic shock^1^ Trend toward sustained restoration of mHLA-DR^1^ Pending results from phase Ib trial^2^	Hotchkiss et al. (162) NCT02960854
Tα1	Restore mHLA-DR expression	No adverse events reported in single RCT^1^ Trend toward improved 28-day mortality^1^ Ongoing phase III clinical trial^2^	Wu et al. (163) NCT02883595
MSC	Reduce inflammatory response Decrease lymphocyte apoptosis Increase phagocytic capacity	No adverse events reported in a phase I clinical trial^1^ Ongoing phase II clinical trial^2^	McIntyre et al. (164) NCT02883803

*Tα1, Thymosin alpha 1; G-CSF, granulocyte colony stimulating factor; GM-CSF, granulocyte-macrophage stimulating factor; IFNγ, interferon gamma; MSC, mesynchymal stem cell; NCT, clinicaltrials.gov identifier*.

### Immunostimulatory cytokines

Early clinical trials attempting to simulation the immune system used granulocyte colony stimulating factor (G-CSF) and granulocyte-macrophage colony stimulating factor (GM-CSF) to enhance phagocyte production, function and improve bacterial clearance. A meta-analysis of trials performed between 1998 and 2011 failed to find a mortality benefit of these therapies, though there was some improvement in clinical endpoints such as ICU LOS (157). IFNγ has similarly been used to stimulate the innate immune response in patients with bacteremia and chronic fungal infections (159, 160). Both are being used in patients with sepsis as part of ongoing phase III clinical trials.

IL-7 is potent inducer of the antiapoptotic protein Bcl-2 that has the ability to both stimulate lymphocyte production and reduce apoptosis. A recent multicenter, randomized and controlled phase IIb trial examined IL-7 administration in patients with septic shock and lymphopenia (161). Patients received low or high dosing regimens of IL-7 for a total of 4 weeks or until discharge. These investigators found an increase in CD4^+^ and CD8^+^ T cells, enhanced T cell activation and potentially T cell trafficking as compared to placebo. In this small trial, there was no difference in mortality or rate of nosocomial infection. The drug was well tolerated with minimal adverse events. Cytokine profiles were measured serially over the course of IL-7 administration and there were no signs of patients developing cytokine storm. Also, the effect of enhanced lymphocyte production persisted for weeks following treatment. These promising results will require confirmation in larger studies to determine if therapy is efficacious and without long-term consequences.

### Checkpoint inhibition

Checkpoint inhibitors such as anti-PD1 and anti-PD-L1, improve monocyte and lymphocyte function and reduce apoptosis through disruption of negative cell-cell interactions. These inhibitors have revolutionized management of malignancy and are now first line therapy for many types of cancer (165). However, immune related adverse events (iRAE) with checkpoint inhibition are not infrequent, with rates of grade ≥3 toxicity approaching 7% (166). Early human studies have demonstrated feasibility and reversal of sepsis induced immunosuppression with *in vitro* blockade (146, 147). A phase I clinical trial of the novel PD-L1 inhibitor, BMS-936559, has been completed (162). A phase I clinical trial of PD-1 inhibitor, Nivolumab, is currently ongoing (NCT02960854).

### Endogenous immunostimulatory proteins

Thymosin alpha 1 (Tα1) is an endogenous thymic peptide that regulates the innate and adaptive immune system. Initial studies in sepsis have shown improvement in mHLA-DR (163). Continued study of this agent is ongoing in a phase III clinical trial (NCT02883595).

### Cellular therapies

Mesenchymal stem cells (MSCs) reduce mortality and organ dysfunction in models of sepsis through modulation of the inflammatory cascade, pathogen clearance and promotion of tissue repair (167). Following completion of a phase I clinical trial in septic shock, administration of MSCs appears to be safe (164). Further research is ongoing in a phase II clinical trial (NCT02883803).

## Conclusions

The clinical phases of sepsis are associated with specific and dynamic changes in the immune programming of multiple cell types. Suppressed inflammatory responses to stimulation have been demonstrated extensively in patients with sepsis, however the dynamics, pathogen, and compartmental specificity of these findings requires additional investigation. Primed immune responses have also been demonstrated in animal models of sepsis survival. This cellular program has not been examined extensively in humans and its contributions to sepsis related morbidity and mortality remains unknown. Molecular mechanisms of immune reprogramming in sepsis still require further investigation. In particular the complexities of PAMP/DAMP-PRR interactions, the role of MDSC and T-reg, and alterations in the epigenome are prime targets for evaluation.

Patients at higher risk for nosocomial infection and mortality frequently experience an immunosuppressed state characterized by defects in immune tolerance, exhaustion, and apoptosis. While reversal of these immunosuppressive phenotypes has improved outcomes in animal models, a direct causal relationship between sepsis-related immunosuppression and nosocomial infection/death has not yet been established in humans. Furthermore, rates of nosocomial infection and its attributable mortality in sepsis may not be as high as previously estimated, suggesting that reasons why septic patients die despite best supportive care still need to be explored. Regardless, nosocomial infections are common and carry such significant morbidity that sepsis patients may benefit from immunostimulatory therapies. Early stage trials of immune therapy have shown reversal of leukocyte dysfunction and good safety profiles, both promising for the potential future of these therapies. However, as sepsis has long-term consequences that are still not well understood and immune therapies may have lasting effects, long-term outcomes of patients receiving immunostimulatory therapy require attention.

## Author contributions

All authors listed have made a substantial, direct and intellectual contribution to the work, and approved it for publication.

### Conflict of interest statement

The authors declare that the research was conducted in the absence of any commercial or financial relationships that could be construed as a potential conflict of interest.
